# Answering 20 more questions on COVID-19 (March-April 2020)

**DOI:** 10.7189/jogh.10.020102

**Published:** 2020-12

**Authors:** Igor Rudan

**Affiliations:** Centre for Global Health, Usher Institute for Population Health Sciences and Informatics, The University of Edinburgh, Scotland, UK

On the 7^th^ of March, 2020, I completed my first editorial on COVID-19, answering the initial 20 questions that arose during January and February [1]. In the meantime, many new insights were published and the events evolved quite rapidly. The public’s understanding of the COVID-19 pandemic has also increased. Thanks to this, I can now offer answers to 20 more questions that have arisen during March and April. This article is reflective of the state of scientific evidence on the 1^st^ of May, 2020.

## 1. WHAT WERE THE EXPECTATIONS OF THE LIKELY DEVELOPMENT OF THE COVID-19 PANDEMIC IN EARLY MARCH 2020?

The developments by the beginning of March were quite similar to those seen previously with SARS and MERS. In both of those epidemics, the primary focal point was decisively suppressed. Then, in both previous incidents, the further spread of the virus was stopped in more than 25 countries using the first line of defense, ie, identifying all those infected, tracing their contacts, and isolating them [1,2].

By the end of February 2020, it was already clear that COVID-19 would also be successfully suppressed in its primary focal point − Wuhan. Then, it was also stopped using the first line of defense in another thirty Chinese provinces, all of which are very large population-wise. The surrounding countries in Asia, ie, Japan, Taiwan, South Korea, and Singapore, seemed to control the virus successfully. Then, on the 24^th^ of February, the first estimates of death rates were published, suggesting that they were much lower than those of SARS and MERS [3].

Furthermore, epidemiologists expected that there would likely be many asymptomatic cases and mild forms of the new disease. Minding the size of Wuhan’s population where those cases would have been unaccounted for, it was still quite possible that the net effect of this virus may not be much worse than of harsh influenza for which no vaccine was available. This is why experts were rather relaxed at this point. At that time, it seemed to them that the scenario of the SARS and MERS was repeating and that the illness itself was not too dangerous. It was reasonable to expect that the epidemic could soon be stopped.

As a result, the World Health Organisation delayed the declaration of a pandemic until the 11^th^ of March [4]. Global stock markets increased by about 10 percent from the 27^th^ of February to the 3^rd^ of March [5]. But for any new and unknown virus, premature relaxation is very dangerous, as will be shown later with COVID-19. By this time, no-one had the opportunity to monitor and study its free spread, nor to determine the speed of the spread. This is because the virus was suppressed by a strict quarantine in Wuhan soon after its presence was realized and confirmed. Everywhere else, it was controlled through the first lines of defense.

## 2. WHAT WERE THE OPTIONS THAT COUNTRIES FACED IN MID-MARCH 2020 WITH RESPECT TO THE RESPONSE TO THE PANDEMIC COVID-19?

When the crisis caused by the new coronavirus from Asia began to spread to Europe and the Americas through air travel, the virus managed to penetrate beyond the front lines of defense in highly developed Western countries. I described the cascade of events that led to the free spread of the virus in Europe in another article [6]. At that point, leaders of these countries were faced with two very unattractive options. Actually, both choices seemed so poor that they found it difficult to judge which one was worse.

With the first option, the reaction was to be instinctive. Nations would be pulled into strict quarantines to prevent the virus from spreading rapidly through exponential growth and the move would protect human lives. During the quarantine, scientists would need to learn more about the virus and assess the level of danger and the effectiveness of different containment measures. But it was clear to everyone that quarantine was unsustainable. It would be enormously damaging to society if enacted without a feasible exit strategy. Namely, after several weeks of quarantine, many people would be left without means for the further purchase of basic groceries. They would also start losing their jobs.

Therefore, the “large quarantine” strategy was undermined by its unacceptability to many people after several weeks. It was going to be increasingly difficult to expect that people would adhere to the level of strict social distancing required to keep the epidemic under control. This would be especially true knowing that the risk of dying from COVID-19 was not particularly high. Then, if many people started ignoring anti-epidemic measures and went back to their pre-COVID-19 lifestyles, many would get infected soon thereafter. Countries would then need to start a new cycle of isolation because their health systems would be unable to handle large numbers of infected people with a severe form of the disease.

Another option for the rulers of western countries was to let the virus spread and infect a significant portion of the population. Under such a scenario, it was hoped that many citizens would gain more permanent immunity by overcoming the disease. As a result, the virus would have fewer people to infect. Its spread would then slow down. However, since neither drugs nor vaccines against the novel coronavirus were available, this approach would inevitably lead to many deaths − especially among the elderly and those with pre-existing diseases. Still, life as we knew it would continue and the economy would be saved from a major disruption.

Yet, the “free spread” strategy was also undermined by the predicted human behavior. Namely, even if the plan were carefully explained to the population and it gained support from a large majority, the rapid increase in the death toll, especially in comparison to quarantined countries, would soon become unacceptable to most of the population. In this scenario, too, it would be increasingly difficult to maintain the responsibility and discipline required to preserve the economy. When people realize that they might end up in intensive care units themselves, many would find excuses to take to their homes and stay isolated for a long time. Then, many businesses would collapse and the number of unemployed people would increase sharply, despite the fact that this plan initially sought to save jobs.

In conclusion, in both possible approaches to this crisis, the behavior of the population, which will increasingly decline to participate in either plan, would eventually undermine the implementation. In the former case, they would resent the plan for economic reasons; in the latter, for fear of their lives and the lives of their families.

This was quite clear to the leaders of the western countries, which is why some were keen to seek their own, third approach. But the approach that would be even worse than these two extreme scenarios was to initially confront the epidemic with one strategy and then change it after a few weeks and move on to the alternative approach. Consistent adherence to the first solution would at least save the maximum number of human lives, albeit only temporarily, but it would significantly harm the economy. Conversely, consistent adherence to the alternative solution would preserve the economy and lifestyle, at least in theory, but with significant loss of life that was extremely difficult to estimate at the time. But, if the leaders picked one initially, and after a few weeks they had to change their minds and choose the other, then both the economy would suffer and the casualties would be quite large. So, governments were facing two seemingly very bad solutions, and the alternation between them would be even worse.

## 3. WHAT WERE THE UNCERTAINTIES AND APPARENT PROBLEMS ASSOCIATED WITH EACH OPTION?

To make the choice even harder than it had seemed already, it had to be made under major uncertainties over nearly all key parameters. The leaders could not be entirely sure what was the case-fatality rate (CFR) of the novel coronavirus SARS-CoV-2 − or its infection-fatality rate (IFR), if there were many asymptomatic cases of infection, which still needed to be established. They did not know the spread rate, either, ie, the R0 parameter. They did know that the growth of cases would be exponential. There was also a major uncertainty over the extent of risk by age and sex. This was mainly because the data from China lumped many age groups together and it was uncertain at the time if COVID-19 may be dangerous for younger people, too [7].

Furthermore, no-one could tell with any certainty if the existing drugs would work against COVID-19, or if they could be repurposed to help save lives. It was not known how long would it take to develop a new vaccine, either. Importantly, no-one could be sure if the acquired immunity would be persistent, or if it would diminish soon. Finally, there was considerable uncertainty whether the virus may be seasonal and simply disappear towards the end of May. These were terrible circumstances to make any important decisions. There were many opportunities to make serious mistakes.

Large quarantines seemed an appropriate temporary solution until scientists could understand how many people become infected without developing significant symptoms. Without that information, it was not possible to predict the effects of the virus on population-level mortality. Although most epidemiologists believed that those effects should be considerably smaller than they appeared from simply dividing the deaths by the confirmed infections, there was no firm evidence for this. The virus was new, so no assumptions were allowed, as they could be very costly and lead to many casualties.

In addition to uncertainties over the “death rate” of the virus, the other uncertainty that worried many epidemiologists was that it “spread rate” remained unknown. If very high, it could make the indirect effects of the new virus worse than the direct ones. It could generate many severe cases that could not be helped due to a health system overload. The consequent overload of the health system would indirectly lead to further casualties due to the inadequate treatment of all other diseases. For this reason, it was extremely difficult to estimate the effects of the free spread of the virus and the number of likely casualties. At the same time, the negative effects of quarantines on human mental health and well-being were also expected. The consequences of economic hardship for many people over the years to come were also very difficult to assess.

The strategy of allowing the free spread of the virus was also very dangerous because it was impossible to predict the ways in which the virus could mutate after infecting so many human carriers. Some new strains could, in principle, become more severe than the current one. A further danger of this strategy was that even low levels of risk for young people would translate into very large numbers if millions were infected. Relatively young victims would surely become a central focus of reporting on the epidemic and undermine any support that this strategy may have enjoyed beforehand.

Another problem with the “free spread” strategy was that if it succeeded to some extent, even with considerable casualties, the virus would still continue to mutate. In its new circle around the world during the next season, COVID-19 would likely shorten many lives again. The immunity acquired during a previous season may no longer protect − as is the case with the flu. This would make many victims of the “free spread” strategy during the first wave seem futile as any “herd immunity” that was acquired then would be lost. Few leaders would consider accepting responsibility for such outcomes in view of so many uncertainties.

## 4. WHAT WAS THE MOST RATIONAL APPROACH IN VIEW OF SO MANY UNCERTAINTIES, THAT WOULD MAXIMIZE THE LIKELIHOOD OF LIMITING THE DAMAGE AND MINIMIZE THE CHANCE OF A MAJOR FAILURE?

Most leaders agreed to quickly and decisively close off the pathways of further spread to the virus. This avoided the unbearable situation of having too many infected people requiring hospitalization too quickly. It protected the health system from collapse and saved many human lives. After that, there would be a very wide range of further options.

The author Tomas Pueyo outlined a sensible strategy of response in Medium.com [8]. It became popular as “The Hammer and the Dance.” The governments of many countries resorted to some variant of this strategy. It was reasonable as it protected people’s lives and the health system, but it also tried to protect the economy as much as possible. The ‘’hammer” was an intensive lockdown, as brief as possible, that would reverse the flow of the epidemic and reduce the number of infected. The ‘’dance” would then be the next phase − our coexistence with the virus − where we would not allow it to spread quickly to a large number of people.

Furthermore, “the Hammer” phase would buy more time for key decisions. During the lockdown we could all learn more about the virus, educate the population, progress some key research, strengthen the capacity to deal with the virus after the lockdown, and learn from others who were ahead in time (eg, Asian countries).

## 5. HOW WOULD THE SUCCESS OF THE COUNTRIES IN RESPONDING TO THE INITIAL WAVE OF THE PANDEMIC BE JUDGED?

This was quite well understood from late March, once it became apparent that many countries were taking proactive measures and implementing lockdowns. The assessment of each country’s performance in dealing with the coronavirus crisis would then be based on the following five questions:

- How effectively did the “first line of defense” manage to prevent the free spread of coronavirus among the population, and for how long?
- Once the “first line of defense” was broken and an exponential spread of the virus began, how quickly and decisively was a lockdown activated?
- How closely did the population adhere to lockdown measures? This step was one where countries would depend on the relationship between the leaders and their population, trust of people in their government’s ability to deal with the crisis, people’s discipline and adherence to measures.
- How fast and active was the state to mobilize its capacities and human resources, as well as creative and innovative solutions, to develop a concrete plan for quarantining and plans for later coexistence with coronavirus as quickly as possible? This needed to include empowerment of technological capabilities and human resources for virus testing, innovative ideas on social removal measures, effective virus control measures, the use of technologies to understand human contacts and the spread of viruses, etc.
- How effectively, after quarantine, has the state enabled its population to move into relatively normal life and preserve their economy from collapse, with permanent control over the spread of the virus?

## 6. WHAT ARE THE COMMON TYPES OF STRATEGIES THAT CHARACTERIZE THE RESPONSES OF MOST COUNTRIES TO THE SPREAD OF THIS NEW CORONAVIRUS INFECTION?

We have seen four different types of strategies. I will rank them from the least to the most interventionist. The first type of response was to allow the virus to spread among the population. The idea was to partly expose the population to the virus. If we could “immunize” ourselves while preserving reasonably normal life, there was a hope that economic activities would not be too disrupted. Sweden, the United Kingdom, the United States, the Netherlands, and, to a certain extent, Germany, led the way in this regard.

The second type of response was to rely on a strong “first line of defense”. In some countries, this approach was facilitated by a high level of economic development, modern technologies, a strong capacity to test people for the presence of the virus, as well as the rapid monitoring and isolation of their contacts with modern technologies. The best examples of this approach were South Korea, Taiwan, Singapore, Japan, United Arab Emirates, Qatar, and Iceland.

The vast majority of countries found themselves in the third group. After a short period in which they tried to stop the virus through their “first lines of defense”, they all had to move to a back-up plan. This involved them closing the borders, schools and banning public gatherings. They were then quarantined for several weeks to prevent the contagion and significantly reduce the daily number of new cases of coronavirus infection. During the quarantine period, exit strategies were being considered on how to co-exist with the virus until effective drugs or vaccines could be made.

Finally, the fourth example involves countries that started with their quarantine measures early enough, or implemented them so intensively, that they practically eliminated the virus from within their respective borders. China was an early example of this, but thanks to the success of its measures so far, there was also hope for New Zealand, Vietnam, Montenegro, and a handful of other countries.

## 7. WHAT HAVE BEEN THE RESULTS OF INDIVIDUAL STRATEGIES SO FAR?

It turned out that all four initial approaches, after enough time, were more or less forced to switch to the same strategy: they needed to apply either a national-level or a localized social distancing of varying degrees of stringency. Although many have sought to preserve their normal lifestyle and indeed their economy for as long as possible, the United States and the United Kingdom now have an enormous number of infected people. The United States is sure to have the highest absolute death toll after the first wave of the epidemic.

The United Kingdom will also have a very large number of coronavirus casualties, which may end up even worse than that USA when expressed per capita. Germany is managing to keep the death toll quite low compared to the number of infected people. Germans owe this to the spread of the infection mainly among younger age groups, as well as excellent epidemic prevention measures in hospitals and retirement homes and their strong intensive care facilities. The Netherlands, like the aforementioned countries, has since moved to stricter quarantine measures [9].

Only Sweden continues to rely on the discipline of its citizens and somewhat less stringent social distancing measures to try to keep the virus at bay. They are trying to avoid a complete lockdown, although some social distancing measures are in place and the population has largely moved indoors. Currently, the death toll is significantly higher in Sweden than in their neighboring countries, but it is obviously not yet considered to be large enough to trigger a complete lockdown [10]. I write more on “the Swedish approach” in my response to question 17.

Unfortunately, we are increasingly seeing that even those nations that have sought to defend themselves against the COVID-19 epidemic by maintaining a “first line of defense” are slowly yielding. For ten weeks, they have been fighting hard to prevent the virus from spreading freely among the population. They achieved this through mass testing and the rapid isolation of patients and their personal contacts. Although it seemed for a long time that these countries would succeed in this approach, Singapore recorded as many as 623 new cases on the 17th of April, mainly among their migrant workers, and a real epidemic is now gathering pace there [11].

The same is true for Japan with 556 new cases of infection, Qatar with 560 and the United Arab Emirates with 477 newly infected people in just one single day. All four of these countries have now substantially tightened their respective social distancing measures [12].

Only South Korea and Taiwan seem to still be succeeding in the “first line of defense” approach. There, the number of newly infected people is still successfully maintained at a low figure without imposing lockdowns. They succeed in this with a massive amount of population testing and the use of rather intrusive methods of monitoring personal contacts through GPS and mobile telecommunications. These two countries have learned from the SARS experience and they were excellently prepared for this one. They remain the only ones to still be resisting it with their “first line of defense” alone, although Iceland and Cuba have also achieved this to a significant degree [13,14].

Iceland and New Zealand have been able to reap the benefits of their favorable geographical locations. Owing to that, they could, much like China, eliminate the novel coronavirus from their territory. However, the most successful reaction in the world, in general, was probably that of Vietnam, another country which recalls the SARS pandemic very well. Vietnam recorded 63 cases and 5 deaths of SARS back at that time. Today, that country, with a population of almost 100 million, still did not record a single victim of COVID-19 [15]. Admittedly, the same may be true of North Korea, but it is quite difficult to know what is happening there with sufficient certainty.

However, a new challenge for all countries that have managed to completely suppress the spread of the virus at an early stage is that, in the next phase, every person entering the country will be a new potential source of the epidemic spread. Therefore, they will only be able to relax their measures when medicines or vaccines are developed. Until then, they will continue to depend on the tight control of their borders and 2-week self-isolation for everyone who enters.

A more general conclusion is that nearly all countries across the world were forced, at least over a period of time, to abandon their initial strategies and move to stricter lockdown measures. There are only a few rare exceptions. Elsewhere, the rapid spread of the new coronavirus simply could not be averted using any other means.

## 8. ARE THE QUARANTINE MEASURES JUSTIFIED, GIVEN THE REAL DANGER OF THE VIRUS AND THE DAMAGE TO THE ECONOMY?

To answer this question clearly enough, we should first have sufficient certainty what the real threat of the virus could be at the level of the entire population. We should also fully understand the economic, health, and any other indirect threats from ‘’halting the world” over several weeks. Unfortunately, at the time of writing this article (1^st^ of May, 2020), we still do not know with sufficient precision the answer to either of those questions. Therefore, all countries are making their own decisions in the face of this unexpected situation, which is entirely new to us and still quite unpredictable.

The extent to which all the measures were justified will be carefully evaluated by science for years to come. The epidemiological profession is still trying to assess the real threat of the virus by employing its methods. The parameters of epidemiological models have been changing. Epidemiologists have been refining them for weeks with each new survey conducted. During this time, it is quite understandable that many people became impatient.

In some countries, a specific problem arose: the results of their health care system’s fight to prevent the spread of COVID-19 were so good, that an increasing number of people no longer saw the measures as justified. Some start criticizing the measures openly. They did not understand that they only encountered few victims during the lockdown precisely because the measures were timely and rigorous. In that sense, some countries are slowly becoming “victims” of their own good results.

However, most people in those countries that have experienced a significantly worse epidemic have quickly realized that “preserving the economy” is very much an illusion until the spread and effects of the new coronavirus are controlled. In the world’s largest economic and military power, the United States of America, there was substantial public support for no strict anti-epidemic measures if they would be likely to ultimately harm more than help.

Then, in New York, Detroit, and some other areas many people started to die and hospitals became heavily burdened. The media’s intense focus on the pandemic has forced most of the population to retreat into their homes, regardless of their plans and the recommendations in place. As a result, jobs began to vanish at an extraordinary pace. The number of unemployed people in the United States has increased at a rate likely not seen since the “Great Depression” that began in late 1929, and it may now surpass it. Thus, in the USA, tens of thousands of people will die in the initial wave of this epidemic, and the economy will also receive a major blow [16,17].

However, there are still quite a few fairly loud and articulate calls in the media asking for readiness to pay that price and ‘’immunize” the population if we could all then resume our normal lives as quickly as possible. These voices believe that such an approach will prove to be better in the long run than quarantine and the consequent collapse of the economy.

The premise for such thinking is that in this first epidemic wave, the level of the population exposure sufficient for “collective immunity” against coronavirus and stopping its further spread will be reached. This level, however, depends on the “spread rate” of the virus, ie, its infectivity. Unfortunately, the level of infectiousness of the new coronavirus appears to be somewhat higher than the initial estimates from Wuhan initially suggested. This then requires a higher proportion of the population to get over the infection to reach collective immunity and stop the epidemic.

At very high levels of infectivity, a proportion of the population that needs to become immune will no longer be 50–60%, but perhaps 70% or even more. In the USA, however, not enough people have been exposed to the infection so far. Barely 10% of the total population had been infected there to date [18]. However, in a country of almost 330 million people, this is still a huge number, so the casualties are already very significant.

Furthermore, in the context of population exposure strategies to preserve the economy, one should also have faith that this immunity will be long-lasting. There has not yet been enough time for this to be explored scientifically, let alone clearly confirmed. Finally, it is hoped that the genome of this virus will remain relatively stable. If it mutates from year to year, much like the flu virus does, the immunity acquired the previous year will no longer protect someone the following year. If we do not receive the vaccine or effective medicines by then, we will have to face a large number of new coronavirus victims once again. This would make all the casualties of the first wave of the epidemic that died so that the rest of the population develops “herd immunity” rather futile, as they could have been prevented with the introduction of lockdowns.

To summarise, the COVID-19 pandemic may, in some unforeseen way, be resolved faster than it appears possible at this point. After that, economies may recover very quickly, or they may not. We are yet to observe this in the coming months. But for many decades, the development of the economy has always meant better human health. It also worked the other way around, meaning that the better health of the people also contributed to economic productivity. However, in this unusual and entirely new situation, the concern for public health threatens the functioning of the economy.

Moreover, there is no “theoretical model” of how much life can be reasonably saved, with the sacrifice of a percentage of the economy. Moreover, there are no good estimates as to whether this economic downturn will have worse health consequences than the pandemic itself. However, one of the most important reasons as to why the vast majority of the world’s countries have chosen measures of social distancing to prevent the spread of the virus is because we still know far too little about the virus. It seemed reasonable, therefore, to buy some time. This will give us a much better idea of what kind of danger we are dealing with and what strategies might or might not be effective. Things are changing, quite literally, day-by-day. It is becoming clearer which measures will work and which are less likely to succeed.

## 9. WHAT DOES THIS MEAN FOR THE COUNTRIES THAT ARE IN LOCKDOWN AND WHEN COULD THEIR MEASURES OF SOCIAL ISOLATION BE ABOLISHED?

If we underestimate this virus after we leave quarantine, coronavirus’ free spread among the population will likely occur again. If people start dying of COVID-19 again in large numbers, it is likely that many people would retreat inside their homes as they did initially. Then, it would be difficult to “save the economy”. If it were so easy to get out of this situation, countries across the world would not be resorting to these strict anti-epidemic lockdown measures. In the vast majority of them, national economies are highly dependent on small and medium-sized companies and as such, they are all in a similar situation. Therefore, countries should work as actively as possible to plan their exit from quarantine, but also to do this with a good deal of caution.

It is certainly good that many countries have now shown that they are able to curb the COVID-19 pandemic using anti-epidemic measures. Of the EU member states, by the 19th of April, 2020, Italy accounted for 22 745 deaths, Spain 20 002, and France 18 681, while the UK, an EU member until recently, had 14 576 casualties. Belgium, Germany, the Netherlands, and Sweden had between 1000 and 5000 deaths, and even non-EU member Switzerland was in this class. Portugal, Austria, Ireland, Poland, Romania, Denmark, the Czech Republic, Greece, and Hungary counted their death toll in three-digit figures, as did Norway and Serbia, which are both outside of the EU [9].

Several other countries in Eastern Europe had much fewer deaths after the first eight weeks of fighting the virus, as a result of early lockdowns. This is certainly encouraging is a praiseworthy success for those nations’ epidemiologists and Civil Protection Headquarters. However, if one wants to understand the true state of affairs with this pandemic when countries are performing well, it is a good idea to look first at Singapore, the United Arab Emirates, Qatar, and Japan. These ambitious nations strive to be the best in the world in solving pretty much every task. That is why the state in those countries is a reliable reflection of what can be expected in the best-case scenario. All four of them are currently fighting outbreaks with a free spread of COVID-19 and they have introduced stricter measures of social distancing − something that only a few would have expected two weeks ago [9].

What does this mean for countries that locked down early and successfully? Suppose they all manage to reduce the number of people infected within their borders to just a hundred by their quarantines. Those hundred infected people would not be apparent to anyone when the lockdown is lifted. They would need to be discovered through testing. This would be done based on the symptoms that they would all develop. Unfortunately, if all the precautions are abolished, those one hundred infected may lead to a thousand infected in just one week, and then ten thousand in the next week. This could lead to a new lockdown. This virus is spreading so quickly and so easily, with such a high rate of spread, that it presents us with very difficult decisions to make.

## 10. IF IT IS TOO DIFFICULT TO ASSESS THE EFFECTS ON THE ECONOMY AND OTHER INDIRECT ADVERSE EFFECTS, WHEN SHOULD WE AT LEAST BE ABLE TO DETERMINE THE REAL DANGER OF THE VIRUS? TESTING FOR ANTIBODIES AMONG THE GENERAL POPULATION WAS RECENTLY CONDUCTED IN SEVERAL COUNTRIES. IT SHOWED THAT MANY MORE PEOPLE WERE INFECTED IN COMPARISON TO THOSE WHO TESTED POSITIVE AND WERE OFFICIALLY REPORTED. DOES NOT THIS MEAN THAT COVID-19 IS NOWHERE NEAR AS DANGEROUS AS IT SEEMED INITIALLY?

The danger of a virus in an epidemic depends, in principle, on its two basic characteristics. In my popular articles, I refer to them as the “rate of death” and the “rate of spread”. I do this for clarity because there are other professional names for them.

The “death rate” is calculated by dividing the total number of deaths from COVID-19 by the number of all who were infected with the virus. For the COVID-19 infection, the death rate cannot be as high as 3.4%, which was an estimate quoted by the Director of the World Health Organisation in the early days of the epidemic [14]. In that estimate, the denominator was based only on persons who had reasons to be tested and were then confirmed positive for coronavirus. In doing so, the denominator did not include all those infected who were not tested. We could not say how many of them existed in any population.

We knew then about the Hubei province that more than 2000 died and that the number of deaths divided by the number of confirmed infected was 3.8%, and in the City of Wuhan, it was as high as 5.8% [7]. Based on the experience of previous respiratory outbreaks, epidemiologists did not believe that every twentieth infected person would die. Specifically, infected physicians at Wuhan’s hospitals had a significantly milder clinical picture when compared to their patients with severe COVID-19. This was a sign that patients in hospitals represented only the extreme end of the clinical presentation, ie, the most severe part of the much broader range of symptoms among the wider population. How many more were actually infected on the streets of Wuhan and had milder symptoms? No one knew.

Wuhan is a huge city with 11 million inhabitants. There could have been as many as 100 000 infected at the time, as the first figures used to compute the “death rate” suggested. But there could have been as many as a million. If this was the case, then the first estimates of “death rates” of 3–6% should have been divided by ten. But because of the size of Wuhan, the number of infected could have theoretically been as high as 10 million. If that was the case, then the first estimates of “death rates” should have been divided by one hundred.

Assuming that the number of infected people should be at least several times the number of people who tested positive for coronavirus in Wuhan, this meant that instead of death rates of 3.4% for the world, 3.8% for the Hubei province, or 5.8% for the City of Wuhan, the rates should be much lower. This is because epidemiologists believed that a large number of people who have milder symptoms and had not been tested needed to be taken into consideration, too.

On the 1^st^ of March, 2020, I made a public prediction of an estimate of “death rate” that was between 0.5–1.0%. In doing so, I said that even a “death rate” of 0.5% was actually a “conservative” estimate since those infected in Wuhan could in principle be between ten and a hundred times higher, due to Wuhan’s sheer size. But, if it is indeed less than 0.5%, it’s already in the rough area of the rate of death from more severe flu.

However, until we have a good understanding of what the denominator is, we will not be able to properly assess the risk of this new coronavirus for public health. During April, the scientific community finally began to receive the first reports from the Netherlands, Denmark, Italy, Iceland, and California [19,20]. Researchers at all of these sites have finally begun to search for antibodies in the blood that indicate that people have become infected with COVID-19 and have become immune to the virus without even knowing it. When such a survey is conducted on a sample that should be representative of the population, we say that we are carrying out the studies of so-called “seroprevalence”.

## 11. EFFORTS ARE MADE TO DETERMINE THE FREQUENCY OF PREVIOUS POPULATION EXPOSURE TO THE VIRUS WITH THE DEMONSTRATION OF AN IMMUNE RESPONSE. WHAT ARE THE CONCLUSIONS OF THE FIRST STUDIES CONDUCTED IN SEVERAL DIFFERENT PLACES?

They have confirmed exactly what most epidemiologists have assumed from the beginning. The number of people infected with the new coronavirus was an order of magnitude higher than the number that tested positive in all of the countries where this was determined. Only a fraction of the population had been infected before the lockdowns, less than 10% in nearly all settings. In the California survey, between 2.5–4.0% of the population were infected [19,20]. In those countries where the “death rate” was driven up by numerous deaths in retirement homes, we should expect a death rate of anywhere between 0.5% and 1.5%. But in Santa Clara, California, where this was not the case, a “death rate” may even be lower than 0.5% [19,20].

These new insights also confirm that the messages from the experts, scientists, and the national civil protection staff about COVID-19 from the very beginning of the pandemic have not been inaccurate. There are two reasons, however, why the correct interpretation of the danger of COVID-19 to the public may have seemed distorted at times. The first one is that the general public does not seem to have even an approximate impression of the danger of influenza as an infectious disease. Flu is not “just flu”. Influenza is a disease that claims between 250 000 and 650 000 victims worldwide every year, even with vaccination readily available [21]. In Italy, about 20 000 casualties were attributed to the flu during the 2014-15 flu season, and as many as 25 000 in the 2016-17 season [22]. Over in the US, as many as 61 000 deaths were attributed to the flu in the 2017-18 season [23]. But the impression of the population about the danger of the flu has become so mild that people do not even ask to get vaccinated, although they are free to do so.

Flu is, of course, a completely new and different disease each and every year. The Seattle-based Institute for Health Metrics and Evaluation (IHME), in its latest estimates, predicted that the number of people who may die from COVID-19 in most countries by autumn 2020 is comparable to severe flu, assuming that anti/epidemic measures against COVID-19 have taken place [24]. The peak of the epidemic wave in terms of deaths in many European countries was scheduled for mid-April, and that was exactly what has been witnessed. I hope that this will contribute to the public’s continued confidence in the epidemiology as a profession [24].

Another reason why the comparisons with more severe flu from the beginning of the pandemic appeared to have been wrong was the events in Italy. The way they were portrayed by the media at the time seemed entirely incompatible with public understanding of the flu. However, Italy presented a version of the epidemic spread of COVID-19 where the epidemiological response was largely missing, and the virus spread freely and quickly among many of Italy’s very old people. The situation there was dominated by epidemics in retirement homes and hospital wards. As a result, the number of infected, and thus of the dead, rose very sharply. The mean age of those deceased was more than 80 years, but this was not apparent from media reports. This is why the “death rate” seemed to exceed 5% [6].

This sounded very scary indeed at the time. However, it was not clear at all from the media reports that the virus was spreading rapidly among the population who were most likely to die, which would provide a correct perspective for those figures. Almost 90% of the deaths were in people who were over 70 years old. In the vast majority of those cases, they were already suffering from one or more serious illnesses. This important information was not clearly communicated. The media only reported on the huge number of deaths, and occasionally on isolated younger patients or terrifying individual testimonies [6].

In their early addresses at the end of February 2020, many epidemiologists suspected that the victims would be predominantly elderly people. For most COVID-19 victims, their lives would be shortened by several years, which would be spent at an older age and undergoing treatment for pre-existing illnesses. There is also another important detail that epidemiologists also emphasized in their first addresses, but due to the then lower level of understanding of pandemics, only a few people noticed or understood that point. In the beginning, journalists and the general public were primarily concerned with the question of the “death rate”, and whether the virus could mutate to increase it and kill more people among those infected. At first glance, that does seem to be the most important question. But many epidemiologists kept saying that the virus could also mutate in another way, to start spreading more rapidly. I personally stated this publicly as early as on the 27th of February, saying that I was actually more worried about that possibility.

## 12. WHY IS THE HIGH RATE OF SPREAD OF THE VIRUS (R0) OFTEN MORE WORRYING FOR THE EPIDEMIOLOGISTS THAN ITS HIGH INFECTION-FATALITY RATE (IFR)?

The problem of the rapid spread of the virus, measured through R0 parameter, seemed far less important to the public at the time than the “death rate”. But it presents a greater challenge to epidemiologists when they are planning measures to contain the epidemic than the “death rate” itself does. From an epidemiologist’s perspective, even a very high “death rate” at which we can still monitor and isolate all infected persons and their contacts is easier to deal with than a low “death rate” with a very rapid spread. The best example is this new coronavirus, which has caused us significantly greater problems with its death rate of around 1% compared to SARS, MERS and Ebola, whose death rates were 10%, 35%, and about 50%.

When the virus arrived in Europe, my concern over the speed of its spread has, unfortunately, came to fruition. The virus’ “spread rate” seemed to be underestimated initially. Its rapid spread made the novel coronavirus significantly more dangerous than its “death rate” did. Initial estimates of the R0 coefficient were around 2.2, but in my own calculations the behavior of the virus in Europe was more consistent with the values within the range of 2.5-4.0, if not more [25]. This meant that each infected individual could infect another three to four healthy individuals. There has even been a pre-publication recently claiming that it was 5.7 [26]. It is possible, however, that this estimate is somewhat too high, but values higher than 3.0 would imply a very rapid spread and should not be underestimated.

## 13. IF THE “DEATH RATE” IS NOW ALMOST CERTAINLY AROUND 1% OR LESS, AND IT MAY EVEN BECOME LESS THAN 0.5% IN TIME, DOES THIS MEAN THAT WE CAN END THE QUARANTINE MEASURES AND IGNORE COVID-19, JUST AS WE NEGLECT THE FLU?

Epidemiologists would be very happy if they could answer positively and say that this was the end of a major public health emergency. However, many things related to this pandemic are considerably more complex than typical public communication related to most other issues. Therefore, the conclusion that follows from the reduced estimates of the “death rate” is still not all that simple.

Unlike other diseases that health care systems regularly face, COVID-19 also has a “fourth dimension” − time. It uses this additional dimension to cause problems that we are simply not used to. In medicine, doctors see too many cases of a certain illness at the same time when, eg, the trauma clinic needs to deal with injured people from a large train or bus crash and they do not have sufficient capacity to help everyone. Another example is wartime surgery, where several military surgeons would need to deal with the sudden emergence of a large number of wounded soldiers following an unforeseen battlefield tragedy. But beyond those rare examples, having too many patients with the same life-threatening condition at the same time is not something that medical professionals often need to worry about.

So, let’s suppose for a moment that this relatively small “death rate” of somewhere between 0.5% and 1% for most western populations is really the only danger associated with it. Then, the new coronavirus could be compared to a monster slowly approaching us from afar. We can see increasingly clearly that the monster is physically small, it reaches only up to our waist. So, we begin to relax. We assume that we should be able to knock something that small to the floor and master it. But, we are only looking at it from the front as it approaches. We cannot check what it has behind its back. There, in a dimension we cannot see, this short monster is hiding three metal bars. When the monster finally stands right before us, if we let him out of our sight for one moment, he will be able to hit us over the head with one of these metal bars at an unbelievable speed.

The first problem that the new coronavirus causes us from that fourth dimension, beyond the death rate itself, is its rapid spread. If we allow it to spread freely among the population, it will infect hundreds of thousands from just one infected person in only a matter of a month or two. Even with a small “death rate”, it will soon lead to thousands of severely ill patients who would require intensive care. All those who do not receive care would then die of suffocation. They would die alone in hospitals because their families would not be allowed to the infected wards. The burial conditions of all the deceased would also be limited and quite different from the usual customs. This is not a fate that anyone would wish on an older member of their family, so it should be avoided.

Another big problem is that the new coronavirus has two faces. When it spreads in the community it is relatively harmless, causing mild to moderate symptoms in most people. But, when it enters into a vulnerable population, such as hospital wards, rehabilitation centers, or retirement homes, it spreads at a tremendous rate, because everyone is particularly receptive. The main problem, though, is that the coronavirus “death rate” then increases by at least ten or twenty times. Among such subgroups, it can kill up to every fifth or tenth infected person, depending on how old and sick they already are. In many European Union countries, a considerable proportion of the total number of deaths was caused by severe outbreaks occurring in retirement homes.

The third metal bar this little monster has in its arsenal is the indirect effect of the virus on the health system, which causes mortality from all other diseases to increase. Specifically, if the virus was simply allowed to spread freely, hospitals would temporarily be crowded with infected patients, doctors, nurses, as well as people who come for check-ups or visits. Many health care professionals would end up either on sick leave due to COVID-19 or in self-isolation so as not to spread the infection to patients in serious conditions. Many patients would then be moved out of hospitals for home care. A disruption of the health system would cause those with other health conditions to die more frequently.

These are the three big problems that no one has a clear answer to yet. This is also why more or less everyone has resorted to a lockdown. The virus is still among us and we have seen at what speed it will continue to spread if we allow it to do so. The first lockdown allowed time to plan the support for the economy, too. However, a second lockdown would really hit the economy hard and frustrate tourism and many other businesses globally.

## 14. IN VIEW OF MANY AMBIGUOUS RECOMMENDATIONS AND FAKE NEWS, WHAT PRACTICAL ADVICE CAN BE GIVEN TO PEOPLE DURING LOCKDOWNS?

First, not all uninfected people are in equal need of protection. The most important group of uninfected people are immunocompromised people − hospital patients in transplant wards, patients with chronic lung or kidney disease, diabetes or cardiovascular disease, especially those with high blood pressure, patients with malignant tumors receiving radiation or chemotherapy, as well as anyone over seventy years of age [27].

They should be particularly protected from infection if they already have a chronic underlying illness and if they live together in large numbers in retirement homes. These are uninfected persons who should really avoid contact with infected persons. In this next phase of the fight against COVID-19, the successes of individual countries will be measured first and foremost by how well they have managed to protect their hospitals and retirement homes.

Then, most of the ambiguities after the lockdown were related to whether or not people could leave their homes, sing on windows and balconies, whether they should wear masks, gloves, and how to dispose of shoes; and whether or not we should disinfect packaging from grocery stores.

We are being confronted with a virus that has shown that it spreads rapidly and very successfully among humans, and it was not yet clear how. Such viruses are usually transmitted by droplets of exhaled air, but also by fingers after someone who is infected touches their eyes or their mouth and nose. We can expect the presence of the virus in tears, mucus from the nose, and in saliva. Another person may inhale it, but also touch the location or object that an infected person has previously touched.

What does this mean for the issue of leaving homes? Theoretically, it is possible to breathe in the air where someone has previously coughed or spoken loudly. Also, it is possible to touch the handrail where someone infected had touched it before. The same is true for door handles. The risk of being infected in this way is, of course, very low, but the transmission of the virus in such a way is not entirely impossible.

Many people need to move and exercise to maintain good health. For this reason, it’s difficult to make a strict recommendation that would apply in all situations and for everyone. For older people, it is certainly better to stay indoors, while for younger people, it is probably better to accept a low risk, go out, and maintain health.

There were a lot of ambiguities surrounding the issue of face masks. There are several sub-questions that should be distinguished. First, do masks primarily serve to protect us or others? If they serve to protect ourselves, then they need to be special, more expensive masks. But in that case, everyone should wear them. As it is difficult for each country to plan for the procurement of special protective masks for all of its citizens, especially since they also have a shelf life, personal protection masks from infection are procured and kept for health care professionals. They will certainly be exposed to infected people. Such protective masks should also be distributed to those who are particularly at risk because of their age or their state of health.

For everyone else, if they were just walking down the street, protecting themselves in such situations is not as important as the above examples from an epidemiological point of view. It is much more important, however, that someone who is infected but has no symptoms does not go on to infect others. Since we cannot know who is infected until they present with symptoms, it is reasonable for everyone to cover up their mouth and nose in some way so as not to inadvertently spread the infection to others.

So, whatever can be done to make it more difficult for the virus to jump from one person to another, should be done. The reason why in many countries there is no strict recommendation on wearing masks is that medical recommendations need to be made on the basis of solid scientific evidence. However, in the case of mask effectiveness, it’s difficult to obtain a sufficient type of evidence when the required outcome of the study should be whether or not there is more or less contamination. When it comes to such research, it would be difficult to grant ethics approval to conduct it.

Therefore, what we know about the effectiveness of masks is based on studies that were not optimally designed, which is why it is difficult to get a firm recommendation from any official body. Epidemiologists believe that masks will help to lower the spread of the infection and that people should cover their nose and mouth so as not to accidentally infect others, especially if they are indoors. They can also use a scarf, a handkerchief, a surgical mask, or anything that would help towards this aim. Common sense suggests that any disruption of the viral spread will help combat this pandemic, even where we do not have the solid scientific evidence we would like in order to make for such a recommendation.

Similar to the question of singing on balconies or going out for a walk, the risk of getting infected through touching packages in supermarkets is probably very small, but until it is better explored, it cannot be completely ruled out. However, those who become infected in this way are likely to have a significantly lower initial entry dose of the virus than via inhaled air. Therefore, the course of the disease may be milder, although science has yet to confirm that hypothesis. But it would be no surprise to epidemiologists if this was proven, although it should always be reiterated that surprises are always possible with new viruses.

## 15. WHAT ARE SOME NEW PIECES OF NEWS FROM THE WORLD OF SCIENCE THAT WE SHOULD PAY ATTENTION TO?

A great deal of scientific research on COVID-19 is currently being conducted in a way that would be quite unacceptable to rigorous science under usual circumstances. All work seems to be done at speed, using very small samples that are often not sufficiently representative for the general population. This highlights only some of the imperfections of COVID-19-related research. Many details that must normally be carefully considered in study design are being neglected in the interest of speed. This led to a parallel epidemic of superficial research, seeking to answer the open questions as soon as possible. Unfortunately, a quick-but-wrong answer can do more harm than the one that is obtained more slowly, but which ends up being correct. But decision-making needs are time-sensitive, which should also be appreciated. This is why decisions in this crisis would often be made using suboptimal information.

An additional issue is that just about every piece of published research received immediate and massive media attention globally. This drove researchers to publish so-called “pre-prints”. Those are scientific papers that anyone could write and publish on a platform for pre-publications, without them being subjected to a serious scientific and professional scrutiny through peer-review. Scientists are trained to understand whether those pre-prints are serious research or have major shortcomings. However, journalists and the general public will have a much harder time assessing that. Therefore, we entered a cycle of daily media reports on various “scientific discoveries” which either proved false or unfounded in about a week or two. It’s a shame that so much time and media space was devoted to unfounded reports and results. It was one of the basic features of the “infodemia” that has now taken hold.

One research question worth exploring is whether the virus can return and infect again those who have already had COVID-19 and were negative on their coronavirus tests [28]. This is now being investigated in more detail. It is possible that sporadic reports of such reinfections can be explained by the tests not being reliable enough, or that it may take much longer for the virus to completely disappear from the body than was initially thought. However, if it turns out that those who have already had the disease can become infected again very soon after getting over COVID-19, that would be very unfavorable. It would mean that the acquired immunity is not permanent and that the vaccine may not be able to help as much as we had hoped. But, it is still too early for such conclusions.

Furthermore, it is becoming less likely that the virus will disappear with the arrival of warmer weather. Recent data from West Africa and many other very hot and humid places show that it is spreading rapidly and successfully [29].

The antiviral drug lopinavir, which works by inhibiting proteases, and together with ritonavir, which is effective against the HIV virus that causes AIDS, has not shown efficacy against COVID-19 [30].

Comparison of the data from China, Italy, Spain, and the US have shown that the new coronavirus has not mutated in terms of a higher “rate of death” or a higher “rate of spread”. Its genome seems to be considerably more stable than that of the flu virus [31].

In addition, there are very early first indications that science has yet to confirm through a proper study design that blood plasma transfusions from those who have recovered from COVID-19, through early-response circulating antibodies, may give a bit of hope to severely ill coronavirus patients. However, the samples which were studied were so small that this line of research needs further work [32].

The most promising story from March and April was the initial efficacy of the antiviral drug Remdesivir. This drug is quite elegant in its approach to prevent RNA viruses from replicating. It is an adenosine analogue and is inserted into the strands of the viral RNA molecule, causing the premature termination of protein synthesis. It was supposed to be a cure for Ebola, but it did not show the desired efficacy against that disease [33].

In the first-ever study among severe COVID-19 patients, which was not designed as a properly controlled trial, it showed possible efficacy in about two-thirds of severe coronavirus patients. The study reported that the death rate of those on respirators decreased from about 50% to 15% [34]. Because of this, Remdesivir should be closely monitored. However, it should be reiterated that the studies conducted on medicines against COVID-19 so far have not adhered to the usual standards. They have been conducted without a control group and we will require much more extensive and better-designed research to properly understand the effectiveness of Remdesivir and other medicines. But in light of all current insights, Remdesivir, with the knowledge of a lower “death rate” in the community, is still the best news we currently have on this front.

## 16. WHY DID EASTERN EUROPE HAVE BETTER RESULTS FROM WESTERN EUROPE IN DEALING WITH THE INITIAL WAVE OF COVID-19 PANDEMIC?

The reasons are quite complex and I explained them in great detail in two separate articles [6,35]. There was a cascade of causes that led to the virus spreading behind Italy’s “first line of defense”, which led to an exponential growth of the number of infected cases. Then, there were several facilitating factors that allowed the virus to spread throughout Western Europe. As an example, ski resorts and UEFA Champions League games were among them, but they were not the only major routes of spread from country to country [6].

Also, the reasons why Italy and other European countries were late with resorting to lockdowns are also complex. They had to do with interactions and communication between politicians, epidemiologists, and the representatives of the private sector. In eastern Europe, it is possible that there was less consideration of “saving the economy”, and more of “protecting the people” during critical decision-making processes that were happening under pressure and great uncertainty described earlier [6].

However, the most important individual factor that determined the epidemiological situation in western and eastern Europe during the first wave of the pandemic was the pattern of population mobility. Western European countries that had lots of air travel with China and also heavy traffic in all forms between themselves in February and early March had a lot of infected cases imported. At this time of the year, countries of eastern Europe had considerably less traffic through their borders. Wherever there was more international traffic, there was more virus imported, and vice versa. Then, the key factor of the eventual success was the response. If the “first line of response” worked well through February and early March and then the lockdown was introduced timely, the results in containing the epidemic were excellent [35]. If “testing, tracing and isolating” were not implemented stringently and the lockdown measures were introduced late, the result was much worse − there were many cases and deaths. So, the differences between western and eastern Europe can be explained through patterns of population mobility − more international travel and late lockdowns led to tragedies, while less international travel and early lockdowns led to good results [6,35].

## 17. SHOULD THE “SWEDISH APPROACH” BE SERIOUSLY CONSIDERED BY OTHER COUNTRIES?

At this point in time, we can only discuss the epidemiological aspects of this approach. The medium- and long-term consequences on the Swedish society will require time to be properly assessed. To understand the epidemiological context, when a situation arises in Sweden where the involvement of experts is required, politics does not interfere in principle. Thus, the Prime Minister left the problem of the COVID-19 pandemic to the state epidemiologist from the National Institute of Public Health, who started planning for achieving “collective immunity though infection” [10].

To many epidemiologists in other countries, this sounded unusual. In principle, “herd immunity” can be achieved by gradual infection. But it is used in public health practice primarily as a principle that prevents mass population infection by vaccination. If everyone needs to be infected to reach that threshold, then “herd immunity” is simply a consequence of mass infection, as if the health care system did not even exist, leading to an unpredictable number of victims [36].

In addition, the proportion of the population that needs to be infected to achieve “herd immunity” depends on the R0 parameter of the new coronavirus, which was not known with sufficient certainty at the time. When R0 is about 2–3, then it is enough to infect about 60% of people. But if R0 is about 5–6, and it could be, then up to 80% or more of people. Therefore, planning for “herd immunity” without a more clearly defined R0 value was actually infecting the population rather blindly − the target proportion was unknown, and so was the number of potential victims.

According to the World Values Survey, Swedes show a unique combination of trust in public institutions and extreme individualism [37]. This is important to remember, as it significantly determines their epidemiological context. They have an unusually high share of one-person households, as much as 40% [38]. With the added, already existing cultural distance between people, this meant that a large portion of the Swedish people was already in a certain kind of quarantine. The government further urged them to exercise caution and suggested maintaining distance. The population of Sweden also has one of the highest levels of personal hygiene in the world [10]. So, it was not easy for the virus to spread in Sweden.

In other EU countries, most cases of the virus spread indoors and within families. Sweden had a big advantage there. Furthermore, Sweden is also known for its immigration programs for highly educated staff from all over the world, in order to balance its labor market. As a result, as many as a quarter of Sweden’s population are relatively young immigrants, most often living in large cities [39]. They became a kind of “buffer zone” for the virus there, because even among them, the rapid spread of the infection did not lead to a significant number of deaths. Due to these three circumstances − the culture of distance, the large number of single people in households, and the large share of young and healthy people in cities − Sweden had a much more favorable epidemiological context, especially compared to the countries of southern Europe.

At the same time, the “death rate” among all infected (IFR) was uncertain. For the new virus, no-one knew what the denominator for calculating the IFR rate really was, because tests to determine the antibody in the blood were not yet available. Events in Italy at the time suggested that the IFR might be as high as 2%, 3%, or even higher. That is why thinking about “herd immunity” without greater certainty in the parameters of R0 and IFR was quite a gamble. An increasing number of scientists and doctors began to protest and put pressure on the Swedish government. As many as 2000 eventually signed a petition [40]. The Prime Minister became actively involved in the events [10].

As of the 29th of March, the Swedish government has banned public gatherings of more than 50 people. Clear recommendations have also been introduced for people over the age of 70 not to leave their homes if they can avoid it. All who can are invited to work from home, avoiding all trips that are not necessary, and tend to be outside the house only when they are not crowded. Sanctions were indicated for non-compliance with these measures [10,41]. As of the 1^st^ of April population was banned from visiting retirement homes, after it became clear that the virus had already penetrated half of those homes in Stockholm [10,41]. Therefore, from the very end of March Sweden is also practically living in a form of quarantine. Much of the population has since lived in a way that has made it very difficult for the virus to spread. Accordingly, a slowdown in the number of newly infected was observed, which is somewhat slower than in other countries, but still present.

As a result of this controversy in the media, many citizens − especially the elderly − withdrew to their homes and further strengthened the quarantine nature of the “Swedish model”. It should be understood that, after all these events, nothing so different is happening in Sweden from other countries, so we should expect similar outcomes. However, the media of many countries, as well as individuals on social networks, continued to write about the “Swedish model” myth for various reasons and motives. Some saw a good alternative story in this − “alone against all”, or “no social distancing measures are needed”. Others had simply felt anti-lockdown for various reasons, so they promoted the myth of “no measures in Sweden” to undermine the work by epidemiologists in their own countries. But this is just another in an endless series of insufficiently accurate information that this crisis abounds in. Fortunately, reasonable solutions eventually prevailed everywhere, even in Sweden itself.

## 18. WHAT MEASURES WERE CONSIDERED DURING LOCKDOWNS TO ALLOW RELATIVELY SAFE COEXISTENCE WITH THE VIRUS AFTER THE LOCKDOWN?

First, health promotion activities aimed to facilitate broader adoption of ‘’gap maintenance”, ie, keeping a safe distance from each other. Also, people will need to consider covering their noses and mouths, washing hands, wearing gloves, and acting with caution and responsibility. This alone will significantly reduce the viral spread.

Then, testing, tracing, and isolating capacity should be enhanced in each country so that they could follow the good examples from South Korea, Taiwan, Japan, and Singapore, who rely mainly on the strength of their first line of defense. They all work hard to constantly “cut paths” for the virus in its spread. Technological solutions are likely to be developed to quickly identify and isolate infected contacts.

If the virus turned out to be considerably more dangerous than we thought, or if it spread much faster, then we would need further approaches to ensure population safety. If we ever become confronted with a virus that kills many more among those infected, innovative defenses could then even consider “dividing” the population into several separate subgroups to “dilute” the people who are available to the virus. This would turn any nation that chooses this approach into a so-called “metapopulation”. Subgroups would be able to leave homes and work on different days.

This approach is similar to a ship or submarine that is internally divided into bulkheads, so they can protect it from sinking if the hull breaks somewhere. That way, if a considerably more dangerous virus triggered an epidemic within one of these subpopulations, it would not spread to the others. A two-day working week during alternate days for every subpopulation may prevent the virus from spreading day-by-day and growing exponentially. Clearly, this is a measure for the worst-case scenarios and more dangerous viruses. “Metapopulation approach” would still allow nine working days a month, with additional work from home where possible.

The combination of all of these measures: (i) the continuous, active detection of infected persons and their separation; (ii) a “safety net” of about 10 000 people for continuous nationwide testing; (iii) splitting the whole of the population into sub-populations, if the virus proves more deadly than we think; (iv) a range of measures to avoid social contacts, such as banning large public gatherings, recommendations on wearing masks and gloves, and restrictions on travel and quarantine for arrivals from abroad; and (v) various innovative technological solutions, such as applications that inform all residents of the status of infection of people they have in their contact list, all of which seem feasible.

This would probably protect us enough from the virus and allow the vast majority of people to continue, more or less, with life as much as possible. Then, the hope remains that during the warmer weather the viral spread would slow down at least somewhat. People will need to get out of lockdowns as soon as possible, so these plans for post-lockdown coexistence with the virus need to be made now.

## 19. WHAT EXIT STRATEGIES EXIST AND WHICH ONES SHOULD BE RECOMMENDED?

Several exit strategies were being considered in most countries during the lockdown. The first one was “complete virus elimination”. This is clearly not eradication, because eradication depends on all countries eliminating the virus. This no longer seems possible until we get a vaccine, but elimination within the country’s borders is a desirable solution if realistic. It requires early quarantine, prolonged high level of discipline of the population, a strong economy that can withstand the quarantine and geographical location that facilitates future surveillance on virus entry and its control using only the “first line of defense”. This kind of approach could lead to ”corona-free zones” all over the world that could exchange people and goods without any concern over the SARS-CoV-2 spreading. Iceland, New Zealand, and Cuba are ideally placed to consider this strategy.

The second potential exit strategy which is proactive and maintains an upper hand over the virus is building a strong “first line of defense”. This requires a series of measures that would lead to long-term coexistence with the virus, but prevent its explosive spread to avoid quarantine. It proved to be a highly effective approach in Taiwan and South Korea, which are its global champions.

The third possible strategy following the lockdowns is “loosening and tightening / limited lockdowns”. It is a realistic option but it leaves human populations in a subordinate position to the virus. The viral spread will continue to dictate daily lives in all these countries. Still, in the absence of capacity to consider better strategies, this is the only realistic option for most countries globally.

Finally, there is an option that may become increasingly attractive to some countries, which could be called the “liberalization of personal risk management”. If the unfortunate situation of the pandemic prolongs and no drugs or vaccines become available anytime soon, some countries may begin to consider granting the right to its citizens to become infected with coronavirus if they so wish. It would mean letting the adult citizens personally assess the acceptable risk for themselves, as well as for all the people they care for, and then adjust their behavior accordingly to that accepted risk.

This last option would require a very close and trusting relationship between governments and their citizens in which information on the epidemiological situation would be assessed and clearly communicated to the population on a daily basis. Then, citizens would respond to this information by collectively taking more or less risk. If this was applied properly, it could keep the spread of the virus within acceptable limits, while optimizing people’s movement and work according to the risk that is acceptable to each individual and that would respect vast individual differences in circumstances. This would be a more efficient way of coexisting with the virus than the anti-epidemic measures that would be applied to everyone, regarding of their differences in risk.

## 20. THERE SEEMS TO BE MUCH FRUSTRATING NEWS ABOUT COVID-19. IS THERE ANYTHING ABOUT THIS PANDEMIC THAT IS POSITIVE FOR HUMANITY? WHAT ARE THE LESSONS LEARNED?

At the end of the first phase, in which the virus spread initially, and the second phase during which it was slowed down through lockdowns, the novel coronavirus SARS-CoV-2 will likely split the countries into two very broad groups. The first group will comprise countries that prepared very well and reacted early to the epidemic. They focused on managing the epidemic and “crushed the epidemic curve”. This will likely also reduce the impact on their economies. The second group of countries was those that considered alternative options and/or reacted too late, or that simply neglected the problem. Those countries will have high numbers of infected, many deaths, and prolonged lockdown. The impact on their economies is still likely to be substantial. So, the first likely lesson will be that it is better to confront any emerging public health problem, learn about it, and deal with it, rather than neglect it.

Second, perhaps this unusual situation will be a historical reminder to both countries and individuals of the importance of self-sustainability and independence from others. Against the trends that globalization brought about in recent decades, this situation led countries to close borders. Then, they suddenly depended entirely on their local production and realized that they had a problem if they had entirely outsourced it to other countries.

This situation may also lead many people globally who will be left without work to consider moving back to villages and reverse the trend of urbanization. Now that wireless internet can be accessed everywhere, it almost does not matter where any person on Earth actually lives. But if they have their own garden and well, at least they will not find themselves in an awkward situation that many may find themselves in these days.

Perhaps one of the consequences of this crisis will be some new idea of organizing the lives of individuals and countries, based on self-sustainability. This would make them, at least in principle, more robust in the face of a number of possible new challenges of the 21^st^ century.

Coronavirus will cause losses for humanity in 2020 on the one hand, but it will reduce losses on the other. For example, it will reduce the number of traffic accidents, the number of victims of violence, and deaths from polluted air. In addition, until recently there were political fights around every percent reduction in fossil fuel use. Now, all of a sudden, this reduction came in an enforced and massive move. In a rather improbable way, this pandemic at least helps combat humanity’s climate change problem.
